# Retronasal Olfaction Test Methods: A Systematic Review

**DOI:** 10.4274/balkanmedj.2018.0052

**Published:** 2019-01-01

**Authors:** Hüseyin Özay, Aslı Çakır, Mustafa Cenk Ecevit

**Affiliations:** 1 Department of Otorhinolaryngology, Dokuz Eylül University School of Medicine, İzmir, Turkey

**Keywords:** Olfaction disorders, olfaction test, orthonasal olfaction, retronasal olfaction, systematic review

## Abstract

**Background:**

This report produces a bibliographic study of psychophysical tests proposed clinical assessments of retronasal olfaction.

**Aims:**

We review how these tests can be utilized and discuss their methodological properties.

**Study Design:**

Systematic review.

**Methods:**

We undertook a systematic literature review investigating the retronasal olfaction test methods. PubMed, the free online MEDLINE database on biomedical sciences, was searched for the period from 1984 to 2015 using the following relevant key phrases: “retronasal olfaction”, “orthonasal olfaction”, “olfaction disorders”, and “olfaction test”. For each of the selected titles cited in this study, the full manuscript was read and analyzed by each of the three authors of this paper independently before collaborative discussion for summation and analytical reporting. Two reviewers independently read the abstracts and full texts and categorised them into one of three subgroups as follow, suitable, not-suitable, and unsure. Then they cross-checked the results, and a third reviewer decided assigned the group “unsure” to either the suitable group or the not-suitable group. Fifty eight studies revealed as suitable for review by two authors whereas 13 found not suitable for review. The total amount of 60 uncertain (unsure) or differently categorized articles were further examined by the third author which resulted in 41 approvals and 19 rejections. Hence 99 approved articles passed the next step. Exclusion criteria were reviews, case reports, animal studies, and the articles of which methodology was a lack of olfaction tests. By this way excluded 69 papers, and finally, 30 original human research articles were taken as the data.

**Results:**

The study found that the three most widely used and accepted retronasal olfaction test methods are the retronasal olfaction test, the candy smell test and odorant presentation containers. All of the three psychophysical retronasal olfaction tests were combined with orthonasal tests in clinical use to examine and understand the smell function of the patient completely. There were two limitations concerning testing: “the lack concentrations and doses of test materials” and “performing measurements within the supra-threshold zone”.

**Conclusion:**

The appropriate test agents and optimal concentrations for the retronasal olfaction tests remain unclear and emerge as limitations of the retronasal olfaction test technique. The first step to overcoming these limitations will probably require identification of retronasal olfaction thresholds. Once these are determined, the concept of retronasal olfaction and its testing methods may be thoroughly reviewed.

## RATIONALE

### Olfaction: How do We Smell?

Olfaction is a major component of human chemosensation that integrates with the other senses and plays a critical role in environmental perception (1). Its contributions to cautionary behavior, such as perceiving spoiled food, fire smoke or a gas leak, make olfaction a vital sensation. Its interrelation with the sense of taste contributes to our ability to enjoy the savor of food (2).

The human olfactory system can recognize approximately one trillion olfactory stimuli (3). Evolutionarily, four vertical anatomical components (two medial constituents of the olfactory clefts and two lateral constituents of the ethmoid labyrinths) comprise the olfactory nose, and the olfactory mucosa covers only the surface of the roof of the olfactory clefts. With evolutionary advances in humans and higher primates, the importance of olfaction has decreased (4).

Olfaction is initiated when odor particles in the nasal air flow reach the olfactory epithelium and interact with odorant binding proteins, and the sensation is finalized with the conclusion of cortical processing. Thalamic connections underlie the conscious portion of olfaction. The entorhinal cortex and amygdala, which are sections of the limbic system, constitute the emotional component of the sensation (5).

Odorants mainly use two different routes for travel toward the olfactory epithelium, located orthonasally and retronasally. Along the orthonasal route, solutes dissolved in the air pass through the nostrils via the turbinates and eventually reach the olfactory epithelium. The retronasal route, however, requires a retrograde direction starting from the oral cavity, continuing through the nasopharynx and choana and ending at the olfactory mucosa (6). According to Rozin, whereas orthonasal olfaction integrates with the external world, retronasal olfaction reflects the inner world (7). As the orthonasal route serves as the primary source of olfaction, the retronasal route also attracts attention, especially for ensuring the integrity of taste perception while eating. Since anosmic people experience alterations in eating habits and suffer from a decrease in taste bud activity, retronasal olfaction may play a critical role in flavor perception (8).

### Measuring Olfaction

The olfaction test battery consists of electrophysiological and psychophysical tests and measurements. Electrophysiological tests measure cortical neural responses to an odor stimulus and olfaction detection thresholds via electroencephalography. Psychophysical tests, on the other hand, provide qualitative information about olfaction rather than the objective results obtained from electrophysiological recordings and thus are only employed for clinical symptom assesment (9).

Psychophysical tests are used to assess olfactory identification (OI), olfactory discrimination (OD), and the olfactory threshold (OT). Among these, the OT refers to the lowest concentration of odor perceived by the patient. However, OD and OI are assigned within supra-threshold values (10).

Numerous validated orthonasal tests have been reported in the contemporary literature. The University of Pennsylvania Smell Identification Test (10), the Sniffin’ Sticks (11), and the Connecticut Chemosensory Clinical Research Center Test (12) are among the most popular.

### What is the Difference Between Orthonasal and Retronasal Olfaction and Why is it Important?

Electrophysiological, psychophysical, and radiological studies point to the differences regarding perception and processing between the retronasal and orthonasal pathways. The disgusting aroma of a piece of stinky cheese can be sensed as a very pleasant flavor. Rombaux et al. (13) highlighted the contributions of air flow variations to this inconsistency and pointed to the importance of nasal air flow. Using functional magnetic resonance imaging, these two routes were shown to activate distinct regions in the cerebrum. Retronasal stimulation was demonstrated to share the same representation area as the oral cavity.

Declines in olfaction and taste sensation act concomitantly. In most cases, taste sensation problems even precede olfaction-related complaints. Therefore, retronasal olfaction tests were used by many authors to evaluate the olfactory component of sensory perception (7). Despite the fact that olfactory disorders are not rare situations and can worsen with both orthonasal and retronasal olfaction and with taste, improvements in retronasal testing lag far behind orthonasal test methods. There is not only a lack of validation of these tests but also a lack of knowledge about whether OI requires supra-threshold measurements (14).

## OBJECTIVE

The current systematic review focuses on the limited available literature described above, with the purpose of examining what is known about psychophysical testing related to retronasal olfaction, as well as reviewing how such testing is employed and discussing the current methodology and its validity.

## MATERIALS AND METHODS

### Search Strategy

We searched PubMed, the free online search engine accessing the MEDLINE database of typically peer-reviewed literature in the biomedical sciences, for the period from 1984 to 2015, using four relevant key phrases: “retronasal olfaction,” “orthonasal olfaction,” “olfaction disorders,” and “olfaction test.”

### Inclusion and Exclusion Criteria

### Subject investigated and inclusion criteria

Our search strategy returned 131 abstracts. The titles and abstracts of all 131 entries were analyzed by two authors of this review, independently of each other, with a scoring system of suitable/unsuitable/unsure as to whether the articles required further reading and review. Abstracts were considered “unsuitable” or “unsure” if they described a case report or experimental research conducted on animals or if methodology involving olfaction testingwas lacking. According to this scoring system, 58 and 13 entries were deemed suitable and unsuitable, respectively. For the 60 entries scored as “unsure,” a consultation with the third author of this study was performed. The consultation revealed 41 suitable and 19 unsuitable abstracts.

### Quality evaluation, prevention of bias, and data exctraction

According to the bias risk assessment tool recommended by the Cochrane Review Handbook 5.1 (15), two reviewers independently read the abstracts and full texts and categorized them into one of three subgroups as follows: suitable, unsuitable, and unsure. Then they cross-checked the results, and a third reviewer assigned articles designated as “unsure” to either the suitable group or the unsuitable group.

### Additional exclusion criteria

**Figure 1:**
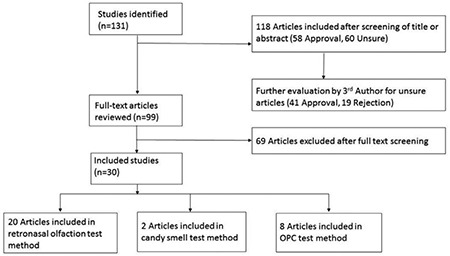
Flow diagram illustrating the study selection process. OPC: Odorant Presentation Containers

Once 99 suitable abstracts were obtained, their full texts were read by the three authors of this study, independently of each other. For these 99 full texts, the following additional criteria were established to exclude articles from further consideration: the article was a review, case report, or study involving animals, or the article included no methodology involving olfaction testing.The authors agreed to exclude 69 of the 99 full texts according to these additional exclusion criteria. Finally, we obtained 30 full texts of original clinical studies. Figure 1 illustrates a flow diagram of the article selection process.

### Intervention and Outcome Measures

The final dataset was searched for psychophysical retronasal tests. Patients’ demographic characteristics; technical and methodological properties of orthonasal and retronasal olfaction tests; and other retronasal-test-related parameters, such as the number of stimulants, the application procedure, subgroup differences, and statistical analyses used to perform validity measurements, were entered and evaluated.

### Data analysis

Outcome measures were recorded in a personal computer. Results were analyzed by using a Microsoft Excel 2010 worksheet (Microsoft, USA) for Windows 7.0 (Microsoft, USA) and are presented in the Results section.

## RESULTS

### Risk of Bias within Studies

According to the bias risk assessment tool recommended by the Cochrane Review Handbook 5.1 (15), all of the abstracts and full-text data were reviewed by the authors of the current study, independently from each other. Our exclision criteria further prevented the risk of bias, because we excluded all studies that did not include a detailed explanation of the olfaction testing method, case reports, animal or experimanteal work, and reviews.

### Results of Individual Studies and Synthesis of Results

The three most widely used and accepted retronasal olfaction test methods uncovered in this systematic review are listed here and detailed below.

• Retronasal Olfaction Test (ROT)

• Candy Smell Test (CST)

• Odorant Presentation Containers (OPC)

### Retronasal olfaction test technique

First introduced by Heilmann et al. (16) in 2002, the ROT is a relatively simple test procedure which assesses retronasal olfaction using an oral stimulant. Taste stimulant powders weighing approximately 50 mg are available in squeezable plastic vials and applied through a 6-cm-long spout placed on the middle of the tongue. Individuals are requested to choose one of four different test agents at each session. Intersession periods last for 1 minute, during which participants are asked to rinse their mouths with tap water. Thirty different food or condiment powders recognizable by 70% of the normosmic population, such as bread, milk, strawberry, ginger, grapefruit, vanilla, onions, oranges, cocoa, celery, coffee, smoked ham, cloves, garlic, white grape, mushrooms, red pepper, lime, raspberry, curry, and cinnamon, were presented to 230 volunteers, both healthy and with olfaction disorders. Substances not recognizable to at least 70% of the normosmic population, such as anise, cumin, bacon, mustard, blueberry, almond, cherry, and coconut, were not employed. Additionally, lemon and pepper, which can be identified by more than 80% of even hyposmic and anosmic patients as a result of trigeminal stimulation, were also not studied. Validation analyses were performed using Sniffin’ sticks TDI score. Results of the comparison between orthonasal and retronasal applications and subgroup differences in retronasal application between normosmic, hyposmic, and anosmic volunteers were statistically significant. Heilmann et al. (16) offered oral flavor powders as useful stimulants and recommended the ROT as an appropriate diagnostic tool.

A year after the study by Heilmann et al. (16), Landis et al. (17) reported using 10 different odorants in a study designed to examine orthonasal and retronasal olfaction in patients with a diagnosis of nasal polyposis. For orthonasal olfaction testing, subjecs were asked to identify 10 substances (lemon, banana, garlic, cinnamon, orange, licorice, apple, mint, pineapple, and coffee), and the Sniffin’ Sticks test battery was used for retronasal olfaction testing. Three substance (rose, turpentine, and leather) involved in orthonasal olfaction and two (fish and cloves) with retronasal olfaction were not included. The application procedure was similar to that of the Heilmann et al. (16) study, and results indicated that retronasal olfaction functions better than orthonasal olfaction in patients with a diagnosis of nasal polyposis. This is most probably related to mechanical obstruction of the anterior portion of the olfactory groove (17).

Retronasal olfaction testing continued to inspire several researchers interested in this issue. Among these researchers, Konstantinidis et al. (18) employed similar testing methods but used different substances, such as oranges, coffee, garlic, cloves, cocoa, celery, strawberries, onion, muscadine grapes, ham, mushrooms, and cinnamon, to understand the possible effects of adenoid hypertrophy (AH) on olfaction and gustation in a pediatric population. As a result of comparing orthonasal and retronasal olfaction between the pre- and postoperative course, adenoidectomy was suggested to have positive effects on retronasal olfaction.

In a study by Pfaar et al. (19) to demonstrate the impact of mechanical obstruction of the olfactory groove on orthonasal and retronasal olfaction, 33 healthy volunteers were tested after having sponge strips placed into the nares on the targeted area, the olfactory epithelium. This research found a significant decrease in orthonasal olfaction compared with retronasal olfaction, with the implication of possible relevance of an orthonasal olfaction defect associated with nasal polyposis.

Subsequently, Rombaux et al. (20) employed the testing technique of Heilmann et al. (16) on a single patient and a sample of healthy participants consisting of four equally sized diagnosis subgroups: nasal polyposis (NP), postinfectious (PI) olfaction deficiency, posttraumatic olfaction decrement, and normosmia. The ROT was adequately capable of appealing the significantly higher retronasal olfaction scores in the NP group.

In 2007, Leon et al. (21) reported a case-control study involving 36 patients who had undergone total laryngectomy (TL) versus 36 smoking control subjects. Orthonasal measurements were performed using CCCRC, and retronasal evaluation was achieved using the method of Heilmann et al. (16). Among 20 variables, another type included grape and chicken bouillon (instead of muscadine grapes and smoked ham) and seriatim, in consideration of incompatible sociocultural taste differences and behavior. It was concluded that both routes of olfaction were hypo-functional in the TL group (21).

Croy et al. (22) carried out a multi-center research study comprising seven different countries and employing the methods described above with some minor alterations. First, 24 of 36 taste substances were used, and second, 20 substances which could be identified by more than 50% of normosmic subjects and by fewer than 50% of anosmic participants were assigned. The results of the research added a significant finding to the literature showing an   age-dependent decrease in orthonasal olfaction, whereas retronasal olfaction was independent of age. Croy et al. (22) asserted that the ROT is not suitable for daily clinical use but should be particularly beneficial in the case of a taste disorder accompanying olfaction deficiency. Studies investigating the ROT are summarized in Table 1, 1-1, 1-2 (16,17,18,19,20,21,22,23,24,25,26,27,28,29,30,31,32,33,34,35,36).

### Candy smell test technique

Renner et al. (37) sought to develop a practical retronasal olfaction testing method that could easily be used in both adult and pediatric populations; hence, the CST was developed. This test makes use of 23 different candies, each including a unique flavor: cola, banana, coffee, lemon, passion fruit, blackberry, cinnamon, orange, pineapple, peach, pear, anise, sweet woodruff (coumarin-like aroma), gingerbread, kiwi, red currant, apple, nuts, vanilla, mandarin, strawberry, mint, and cherry. Five hundred milligrams of sorbitol is used as an inert supplement. The flavors are selected from the Sniffin’ Sticks identifying test. With the nose clipped, the candy is placed on the middle of the tongue, and after sucking on the candy, individuals are presented a choice of one of four different test agents in each session. The main difference between the ROT and this test is the non-stop testing manner during chewing and swallowing of the CST. Each correct answer is scored one point. The cutoff point for anosmia is 13. The test is validated according to the Sniffin’ Sticks TDI scoring, and it possesses 83% specificity and 94% sensitivity (38). Haxel et al. (38) tried the CST in a case-control study, validated it using the Sniffin’ Sticks orthonasal test, and ranked it as an easy and reliable test method that can be performed for discriminating normosmic, hyposmic, and anosmic individuals in daily clinical practice. These studies are summarized in Table 2.

### Odorant presentation container technique

Pierce and Halpern (39) performed pioneering research on finding ways to prevent agents in the oral cavity from reaching the olfactory epithelium. Their discoveries, reported in 1996, first described a procedure in which orthonasal and retronasal stimulation interacted with taste and thermal or mechanical senses while traveling to the olfactory mucosa. The authors developed odorant presentation containers (OPCs) and placed them in the mouth. OPCs were introduced in detail in their original research. Briefly, the specially manufactured OPC is composed of two telescoped cylinders, which provide isolation between the solid odorant in the inner cylinder and the oral tissue. The product also lacks any thermal, gustatory, and taste stimulants. Retronasal olfaction is achieved by OPCs during exhalation through the nose. Visual input is prohibited by asking participants to close their eyes.

Sun and Halpern (40) examined retronasal olfaction using OPCs and cinnamon, anise, mint, coffee, orange, and strawberry as the odorants in their work. A spirometry nose clip was employed to stop orthonasal airflow during the test. Chen and Halpern (41) made further use of the OPC method using non-trigeminal stimulants such as coumarin, octanoic acid, vanilla, octane, and phenyl ethyl alcohol. Studies investigating odorant presentation containers are summarized in Table 3, 3-1 (39,40,41,42,43,44,45,46). Grenn et al. (45) also used OPC with non-trigeminal stimulants using vanilla, citral, and furaneol.

## DISCUSSION

### Summary of Evidence

### Summary of the retronasal olfaction test

A consistent finding among studies that focused on ROT was the strict use of the original retronasal olfaction measuring technique introduced by Heilmann et al. (16). Applying the test powder in the mouth while clipping the nose is a standard practice. To assess the validation of this test, many authors utilized the orthonasal olfaction test SST.

In our opinion, the selection of preferred odorant agents should be made with consideration of their societal commonality. Particularly, the test agents used in retronasal olfaction testing should be varied because of the sociocultural variations seen with orthonasal testing. For this purpose, some authors employ a preliminary questionnaire to learn the most familiar and recognizable flavors (22). A Turkish population survey made by Salihoglu et al. (33) in 2014 required a modification of the test powders because of sociocultural mismatch. After a questionnaire about the knowledge of flavors planned for the test was completed by all participants, celery, curry, ginger, ham, and blackberries were exchanged for cumin, sesame, thyme, sausage, and banana, respectively. The conclusion of this study was compatible with the method of Heilmann et al. (16). The author re-used the test in his later study that focused on evaluating retronasal olfaction in obstructive sleep apnea (OSA). No significant decrease was experienced in retronasal olfaction in contrast to ortanasal olfaction. The authors attributed their result to cultural variation (31). Besides, many researchers consistently excluded lemon and pepper due to their trigeminal stimulant characteristics.

Another interesting issue is that nasal mechanical obstruction, NP, and AH are disorders found to disrupt orthonasal more than retronasal olfaction. However, retronasal olfaction recovers after surgical treatment. Altundag et al. (34) employed the same test to investigate the relationship between AH, tonsil hypertrophy (TH), and retronasal olfaction, indicating that AH was a negative effector in both orthonasal and retronasal olfaction, but TH was only a factor in retronasal olfaction. Correlation analyses showed that the size and volume of adenoid tissue were stronger determinants of retronasal compared with orthonasal olfaction. On the other hand, pathologies that block the upper airway, such as TL, reduce both orthonasal and retronasal olfactory function. Advancing age is, interestingly, only responsible for a reduction in orthonasal olfaction. Despite its frequent use, the retronasal test is only performed within supra-threshold margins in the literature. Thus, the exact threshold concentrations remain undefined.

### Summary of the candy smell test

The CST, like the ROT, utilizes test ingredients that stimulate both taste and smell simultaneously. Additionally, and similarly to the ROT, the intensity, concentration, and doses of applications are undefined in CST. Both tests ignore the proper thresholds of retronasal olfaction.

### Summary of the odorant presentation container test

The OPC method obtains information about the air phase of retronasal olfaction in the supra-threshold zone in addition to taste function. Mechanical stimulation of the oral-cavity mucosa and the unknown concentration of the odorant inside the mouth are the most significant limitations of this technique. Moreover, the contributions of orthonasal olfaction are ignored during this technique.

### Limitations

Our study of the literature consistently revealed two limitations of olfaction testing: a lack of use of known concentrations and doses of the test substances and conducting tests within the supra-threshold zone. Additionally, no particular procedure was described to detect threshold sensation. The absence of such standardizations probably underlies the delay in progress of these tests and prevents them from being employed in routine clinical use.

In conclusion, the appropriate test agents and optimal concentrations for the ROT remain unclear and emerge as limitations of the ROT technique. From our point of view, the first step to overcoming these limitations will probably require identification of retronasal olfaction thresholds. Once these are determined, the concept of retronasal olfaction and its testing methods may be thoroughly reviewed.

## Figures and Tables

**Table 1:**
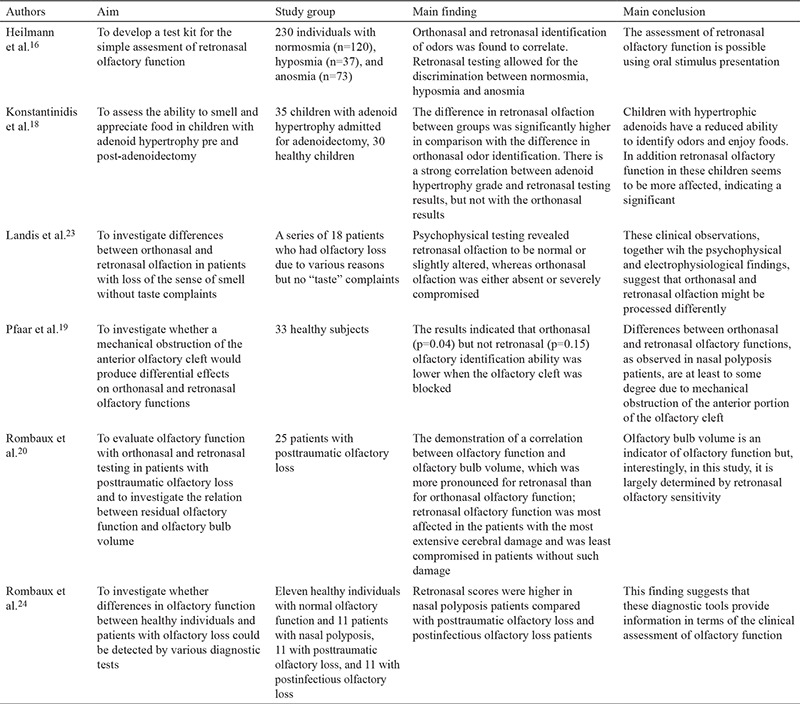
Summary of studies investigating the retronasal olfaction test

**Table 1-1:**
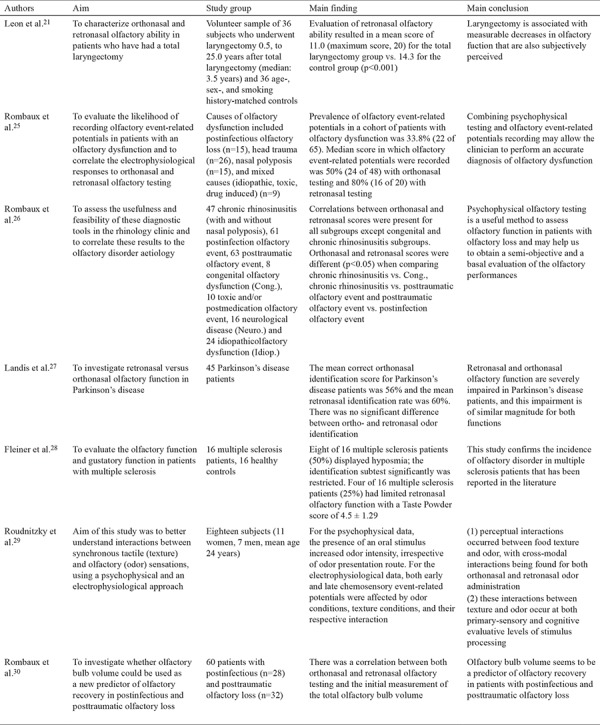
Summary of studies investigating the retronasal olfaction test

**Table 1-2:**
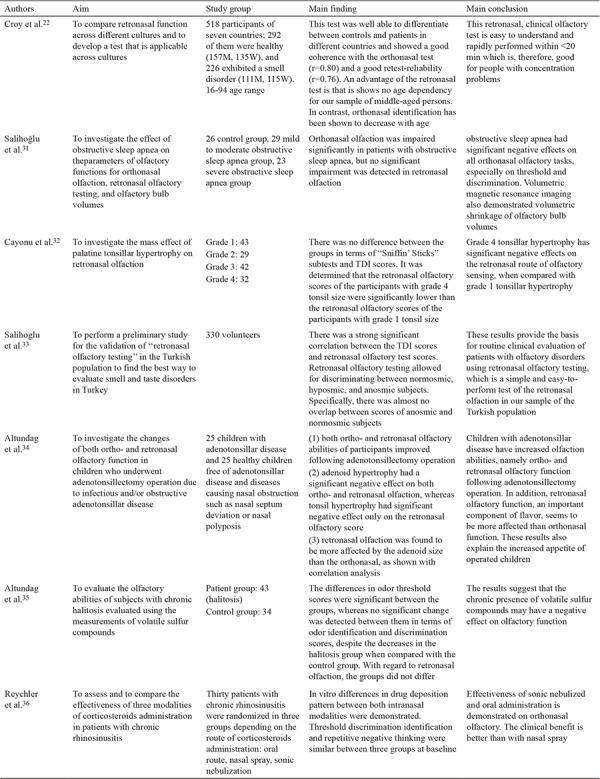
Summary of studies investigating the retronasal olfaction test

**Table 2:**
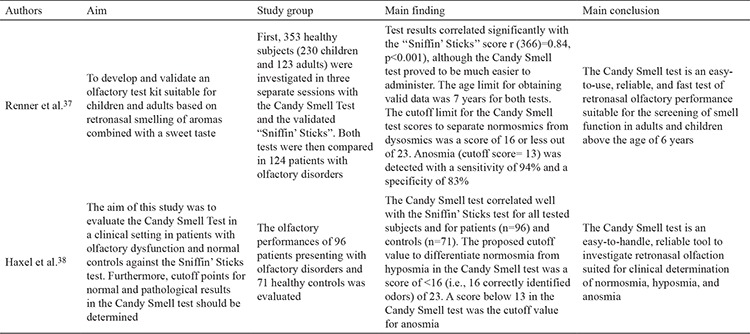
Summary of studies investigating candy smell test

**Table 3:**
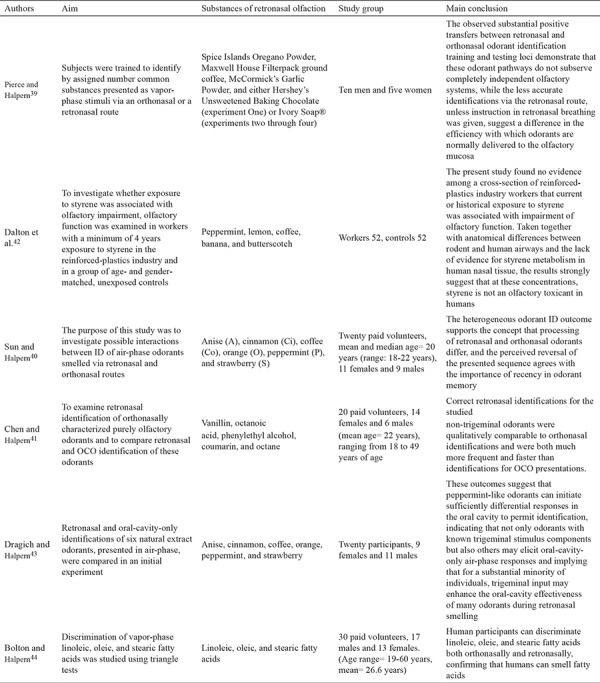
Summary of studies investigating odorant presentation containers

**Table 3-1:**
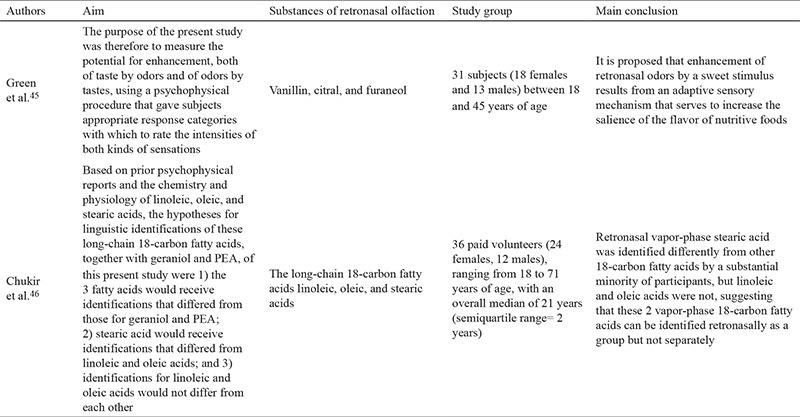
Summary of studies investigating odorant presentation containers
